# Prescribing Immunosuppressive Treatment for Older People with Glomerular Diseases: Time to Assess Frailty?

**DOI:** 10.34067/KID.0000000000000095

**Published:** 2023-03-01

**Authors:** Henry H.L. Wu, Alexander Woywodt, Andrew C. Nixon, Karthik K. Tennankore

**Affiliations:** 1 Renal Research, Kolling Institute of Medical Research, Royal North Shore Hospital & The University of Sydney, Sydney, Australia; 2 Department of Renal Medicine, Lancashire Teaching Hospitals NHS Foundation Trust, Preston, Lancashire, United Kingdom; 3 Division of Cardiovascular Sciences, Faculty of Biology, Medicine & Health, The University of Manchester, Manchester, United Kingdom; 4 Division of Nephrology, Department of Medicine, Dalhousie University, Halifax, Canada

**Keywords:** glomerular disease, frailty, frailty assessment, immunosuppressive therapy

## Introduction

Frailty, the age-associated cumulative decline of multiple physiological systems associated with a disproportionate change in health status when exposed to stressor events, is common in people with CKD, especially in older adults.^1,2^ Frailty in CKD is associated with multiple negative outcomes, including falls, decreased quality of life, hospitalization, and death.^2^ Frailty can evolve over time and is potentially amenable to intervention, as are the associated adverse outcomes.^3^ Formal assessment of frailty has been suggested as part of the transplant assessment and wait-listing,^4^ although the relationship between frailty and post-transplant outcomes is complex.^5^ In comparison, frailty has received much less attention in older people with glomerular disease despite the fact that both groups share important short, medium, and long-term risks related to immunosuppressive medication. Frailty status may provide important prognostic information in the context of glomerular disease that could inform shared decision making. In this study, we compare the current evidence surrounding frailty assessment in the context of glomerular disease with that in CKD and transplantation, highlight areas of uncertainty, and suggest implications for clinical practice.

## Frailty in CKD, AKI, and Kidney Replacement Therapy

In CKD, the association between frailty and poor health outcomes was well characterized in a multicenter, prospective study of people with late-stage CKD.^6^ Frailty was highly prevalent and associated with a two-fold higher risk of all-cause mortality.^6^ Interestingly, participants considered frail by subjective clinical impression had near four-fold higher relative odds of selecting in-center dialysis.^6^ In AKI, frailty is associated with a comparably high risk of death. In a study of critically ill people with severe AKI, a higher frailty severity was associated with a 50% increase in 90-day mortality.^7^ In chronic dialysis, frailty has been associated with mortality, hospitalization, and other health outcomes.^8–12^ Finally, people categorized as frail during wait-listing are less likely to survive to transplant.^13,14^

A growing body of evidence describes the association between frailty and outcomes after kidney transplantation. Frailty has been associated with mortality; early rehospitalization; and other negative outcomes, such as post-transplant infections and graft loss.^15,16^ In a prospective cohort of 525 kidney transplant recipients, frailty was assessed at the time of transplant using the frailty phenotype.^17^ Dose reduction of mycophenolate mofetil (MMF) occurred in 54% versus 45% of frail versus nonfrail recipients. There was a 30% increase in the relative hazard for time to dose reduction (hazard ratio, 1.29; 95% confidence interval, 1.01 to 1.66). In turn, MMF dose reduction was associated with an over five-fold increase in death-censored graft loss. It is possible that similar immunosuppression dose reductions occur in frail individuals with glomerular disease, potentially leading to suboptimal treatment.

## Frailty in Glomerular Disease Treated with Immunosuppression

While formal evaluation of frailty status is not currently a routine part of assessing people with glomerular disease, consideration of whether an individual may be robust enough to tolerate immunosuppression has always been part of decision making. A good example is the treatment of ANCA-associated vasculitis (AAV), where much of recent research has focused on delineating less toxic treatment regimens for older individuals and those with comorbidity. Although frailty and comorbidity are interlinked, a distinction should be made between the two entities: comorbidity as the aggregation of more than one clinically manifested disease state in an individual and frailty as the age-associated loss of reserve across multiple physiological systems (which may be subclinical).^18^ The prevalence of frailty increases with comorbidity, but one can exist without the other. In contrast to comorbidity, frailty has only recently received attention in AAV. A 2020 study by McGovern *et al.*^19^ evaluated a formal assessment of frailty in AAV and demonstrated that baseline frailty was associated with longer index hospital admission and greater mortality. In this study of 83 subjects older than 65 years with AAV, the risk of death doubled with each higher unit of the Clinical Frailty Scale score,^19^ although a subsequent smaller study did not show this association.^20^ The study by McGovern *et al.*^19^ also added to concerns on the adverse effects of cumulative steroid dose exposure in the older AAV population. Although there were no significant differences in the cumulative glucocorticoid dose exposure between the more and less frail groups, there were more adverse events in the frail group (1.4 versus 1.1 events per patient) and a greater proportion of frail group patients had one or more adverse event(s) (81% versus 58%). The most common reported adverse event overall was hospitalization secondary to infection, which is a recognized adverse effect of immunosuppression treatment (the study publication did not separately report the distribution of adverse events by frailty groups). However, clinicians should also be mindful of the risks of undertreating AAV, balancing this with the risks of treatment-related toxicity. A recent meta-analysis demonstrated that older adults with AAV had better survival when receiving induction immunosuppression.^21^ Recent research has delineated a less toxic but equally effective rituximab-based and steroid-reduced (or steroid-free) treatment regimen^22–25^ that could be an effective way of maintaining disease-modifying treatment while mitigating steroid-associated adverse events.

**Figure 1:**
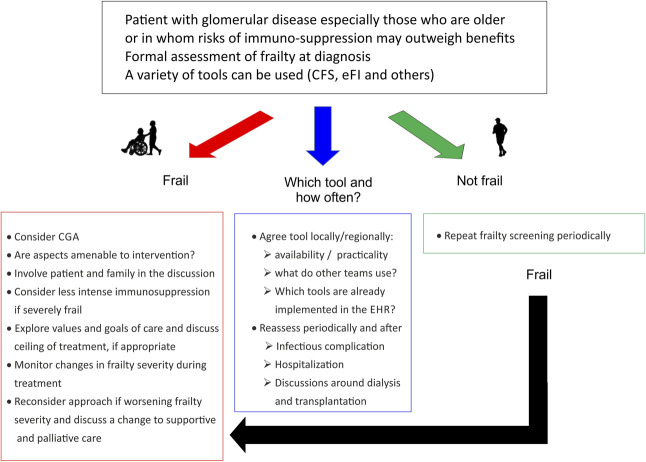
**Suggested approach to formal assessment of frailty in people older than 65 years with glomerular disease requiring immunosuppression.** CFS, Clinical Frailty Scale; CGA, comprehensive geriatric assessment; eFI, electronic Frailty Index; EHR, electronic health record.

Outside of AAV, there has also been interest in using frailty assessments before initiating treatment in nephrotic syndrome. Membranous nephropathy, amyloidosis, and minimal change disease are the three most frequent diagnoses in older adults presenting with nephrotic syndrome.^26^ Frailty status, in addition to age, comorbidity, falls history, and cognitive impairment, may inform clinical decisions surrounding immunosuppressive treatment.^27^ Another interesting question is whether frailty should influence our decision to pursue kidney biopsy, which may place older adults at a higher risk of complications.^26^ We would suggest that most clinicians already take frailty into consideration in this scenario, but there remain limited data on how more formal assessments may be useful in guiding decisions around kidney biopsy. One would expect that a better understanding of frailty and outcomes of immunosuppression will help identify those who should not be biopsied because they are unlikely to benefit from immunosuppressive treatment. Figure 1 describes our proposed approach. This framework offers considerations for adopting a conservative versus “standard” immunosuppression prescription strategy in older patients with glomerular disease on the basis of frailty status. Importantly, it emphasizes the importance of periodic reassessment of frailty status within this context. Shared decision making on immunosuppression treatment in those considered frail or with worsening frailty status can be informed by comprehensive geriatric assessment (CGA), which is a multidimensional, multidisciplinary process that identifies medical, social, and functional needs, alongside reassessment of care goals with patients and their families.^28^ After initial frailty screening assessment(s) to monitor for changes in frailty status during immunosuppression treatment, CGA may be helpful to identify possible contributors of worsening frailty status potentially amenable to intervention and to develop a coordinated care plan to address identified issues. If frailty is considered irreversible, as is likely the case in more severe frailty states, consideration should be given to less intensive immunosuppression strategies, supportive care, and advance care planning. Hampering this field is the paucity of prospective studies evaluating frailty in the older population with glomerular disease, including AAV. There remains no consensus on which tool to use for frailty assessment, with a resulting landscape of multiple competing tools being used concurrently. Apart from the lack of consensus on tools for frailty assessment, there are other issues relating to training and governance requirements when performing frailty assessment in the glomerular disease population, as well as the documentation of assessment findings and education of patients and families. Table 1 provides a brief list of these issues and some suggestions for mitigation.

**Table 1:** Potential issues around the assessment of frailty and mitigation strategies

Potential Issue	Suggestions for Mitigation
Lack of standardized tools to assess frailty	Aim for international consensus on the approach to screening and measuring frailty
International consensus on tools for assessment may not be realistic in the near to mid-term future	Consider using a simplified screening tool (i.e. the Clinical Frailty Scale) as a first step, followed by a more comprehensive tool as resources permit
Effect on workload	Maximize information technology (IT) and leverage infrastructure to allow more seamless capture of frailty status/severity and to develop training programmes to empower staff in the use of tools
Training and retraining	Consider a departmental or institutional approach
Interclinician and interteam variability of assessments	Foster a multidisciplinary and team approach and discussion of patients in whom there is variability around the level of frailty severity
Documentation of frailty assessment	Ensure all IT systems record frailty assessment and that there are measures in place to avoid duplications in documentation
Periodic reassessments of frailty severity	Consider adding scheduled prompts to IT or date by which an updated reassessment is needed
Lack of understanding of frailty as a concept among patients and families	Patient information and educational campaigns
Requirement for transparency	Incorporate frailty assessment in letter templates and share correspondence with patients and providers; use clinical encounters to mention and explain frailty and address concerns from patients and their families
Risk of stigmatizing patients	Education of patients, families, and caregivers
Risk of rationing of resources away from frail patients	Training of staff, audit, and quality assurance

Formal assessment of frailty is increasingly incorporated in the evaluation of people with advanced CKD and in those being considered for transplantation. However, formal frailty assessment is not routinely used to help with decision making and prognosis in older people with glomerular disease undergoing immunosuppressive treatment. The 2021 KDIGO clinical practice guideline update on glomerulonephritis and its treatments contained only a single mention of the word “frail.”^29^ This is particularly noteworthy given the fact that nephrologists have always, either consciously or subconsciously, considered frailty as part of their decision making (although they may not have used the term). There is insufficient evidence to routinely use frailty status as the key factor in immunosuppression decision making at present, and we cannot simply extrapolate from the evidence base in transplant nephrology. Further research should now study whether and how a more formal and detailed assessment of frailty can add prognostic value and help guide glomerular disease management. For now, we encourage clinicians to consider frailty when assessing older people with glomerular diseases and, where frailty is identified, consider broader health care needs and the appropriateness of a goal-directed approach to management.
